# Exploring the relationship between Chinese physical education student teachers’ teacher identity and their interactions with cooperating teachers

**DOI:** 10.3389/fpsyg.2026.1746480

**Published:** 2026-02-16

**Authors:** Fengyu Weng, Shiqi Zhuang, Jiren Zhang, Wei Qiao

**Affiliations:** 1 College of Physical Education, Huaqiao University, Quanzhou, China; 2 Department of Health and Physical Education, The Education University of Hong Kong, Hong Kong, Hong Kong SAR, China; 3 Department of Physical Education, Xiamen Institute of Technology, Xiamen, China

**Keywords:** cooperating teachers, interaction ritual chain theory, student teachers, teacher identity, teacher-student interaction

## Abstract

**Purpose:**

The teaching practicum constitutes a pivotal stage in the preparation of preservice physical education teachers (PPETs), during which their experiences exert a profound influence on the formation and development of teacher identity (TI). This study explored the relationship between PPETs’ TI and their interactions with cooperating teachers during the practicum, drawing on Interaction Ritual Chain (IRC) theory as an interpretive framework.

**Methods:**

A total of 944 physical education student teachers (552 males, 58.5%; 392 females, 41.5%) participated in the study. Both cooperating teacher-student teacher interactions and TI were measured through standardized questionnaires. The data analysis involved two stages: Pearson’s correlation was first employed to identify bivariate associations between the dimensions of cooperating teacher-student interactions and PPETs’ TI. Subsequently, multiple regression analyses were performed to determine the extent to which specific interaction dimensions predicted TI.

**Results:**

Analyses showed that cooperating teacher-student teacher interactions were positively correlated with and significantly predicted TI. Shared activities, bodily co-presence, and emotional resonance emerged as key factors, while a supportive interactive climate and diverse approaches further promoted PPETs’ TI development.

**Conclusion:**

These findings suggest that the quality of cooperating teacher-student teacher interactions play a crucial role in fostering PPETs’ TI.

## 1 Introduction

Teacher education has been widely acknowledged as one of the most influential levers for improving educational quality worldwide (Ambu-Saidi et al., 2024). Research consistently shows that teacher effectiveness is a key driver of student learning (Ye et al., 2024). It further underscores that the foundation for such effectiveness is laid during the professional preparation phase. In this regard, the concept of TI has emerged as a central theme in the study of preservice teachers’ professional development (Jiang, 2012; Li et al., 2022). TI encompasses how preservice teachers see themselves, how they are recognized by others, and how they negotiate the tensions between personal beliefs, institutional expectations, and classroom realities (Rushton et al., 2023). The prevailing understanding has shifted from viewing it as a fixed attribute to recognizing it as a dynamic, socially constructed process that evolves throughout teacher education and professional practice (Solari and Martín Ortega, 2022).

Unlike other subjects, the very nature of physical education is defined by an embodied and movement-based learning environment, creating a set of pedagogical, social, and organizational demands unique to the discipline (Rocliffe et al., 2024). Their work often requires them to balance health-related fitness promotion (Yang et al., 2024), skills teaching (Dudley et al., 2012; Lin et al., 2022), classroom management in physically active settings (Amorim and Ribeiro-Silva, 2022), and the cultivation of positive attitudes toward lifelong physical activity (Duggan, 2022). For PPETs, the process of becoming a teacher thus involves not only acquiring pedagogical knowledge and practical skills, but also negotiating how they understand their professional roles in environments that are inherently public, performative, and relational (Ward, 2013; Athanases et al., 2020; Cho et al., 2024). The development of a strong and resilient TI is therefore critical to their persistence in the profession and to their ability to fulfill the broader educational missions of PE (Simon and Weissblueth, 2024).

### 1.1 Factors influencing PPETs’ TI

Through a review of the existing research, it was found that PPETs’TI is influenced by a variety of factors (Yang et al., 2023), such as the teacher education program (Puchegger and Bruce, 2021; Simon and Weissblueth, 2024; Yang et al., 2026), self efficacy (Rubio-Valdivia et al., 2024; Sun et al., 2025), body image or physicality (Virta et al., 2019; González-Calvo et al., 2022), past experience (Barber et al., 2022) and student teaching (Virta et al., 2023; Güler and Tuncel, 2025). Among these, student teaching is often regarded as the most decisive for identity construction (Huang et al., 2023; Yang et al., 2023). It represents a pivotal stage in the transition of PPETs into in-service professionals, and virtually all PETE programs worldwide incorporate this component (Azevedo et al., 2023; Ward et al., 2023). Numerous studies have underscored the significance of student teaching in shaping PPETs’ TI. Margarida et al. (2012) examined the daily experiences of a student teacher and found that, within the practicum context, interactions with colleagues, students, and other teachers played a crucial role in shaping her understanding of what it means to be a teacher (Margarida et al., 2012). Similarly, Kim et al. (2026) demonstrated that the teaching practicum plays a pivotal role in the formation of PPETs’ teacher identity, showing that student teaching provides a critical context in which preservice teachers’ beliefs are enacted, emotions are elicited, and an initially vague or fragile sense of teacher identity can be progressively consolidated through lived teaching experiences (Kim et al., 2026). Student teaching also represents the point at which preservice teachers begin to reconcile theoretical knowledge from coursework with the lived realities of classroom practice (Kaya, 2023; Phillips and Condy, 2023). The construction of TI is a complicated process. Previous studies have shown that student teaching can either strengthen or destabilize preservice teachers’ emerging identities, depending on the kinds of experiences and interactions they encounter (Trent, 2010; Yuan and Lee, 2016). For PPETs, who must navigate both the technical demands of teaching physical skills and the relational challenges of managing diverse groups in active settings, the practicum is particularly consequential (Yang et al., 2023).

However, some studies present an alternative perspective, suggesting that TI is seldom directly shaped by school placements. Instead, experiences prior to entering initial teacher education programs may exert a stronger and more lasting influence on identity development (Amorim and Ribeiro-Silva, 2022; Amorim and Silva, 2025). These studies are largely qualitative in nature, and their findings may be affected by differences in national contexts, cultural backgrounds, and individual experiences, which may account for variations across studies. Importantly, this perspective does not negate the role of student teaching. Rather, it suggests that the practicum often serves to reinforce or fine-tune pre-existing TI rather than acting as the sole determinant.

### 1.2 The role of cooperating teachers in student teaching

Within the practicum context, cooperating teachers play roles as supervisors, mentors, and evaluators (Nesbitt et al., 2022; Bibi et al., 2025). They are regarded as “significant others” in the professional learning of student teachers because their feedback, modeling, and interpersonal support play a formative role in shaping novices’ perceptions of teaching and of themselves as teachers (Beshears et al., 2021; Chien, 2022). Research has demonstrated that supportive and collaborative mentoring relationships can enhance student teachers’ confidence, encourage reflective practice, and facilitate positive identity development (Bastian et al., 2022; Mwesigwa and Nakato, 2026). Conversely, mismatched expectations, lack of feedback, or overly critical supervisory practices can lead to disillusionment, anxiety, and even the erosion of TI (Derakhshan and Nazari, 2023). Beyond supervision, cooperating teachers serve as professional models, demonstrating effective teaching strategies, classroom management, and the integration of theoretical knowledge into practice (Baco et al., 2023). For PPETs, whose practicum involves both teaching physical skills and managing active, diverse groups, cooperating teachers are especially influential. Their guidance not only shapes practical teaching skills but also supports identity negotiation, helping preservice teachers reconcile professional ideals with lived classroom experiences (Amorim and Ribeiro-Silva, 2024). In this sense, student teaching functions as both a site of skill acquisition and a critical arena for professional identity development, with cooperating teachers playing a decisive role in this process.

Although existing studies have highlighted the important role of cooperating teachers in supporting the professional growth and skill development of student teachers, it remains unclear whether and how the interactions between cooperating teachers and student teachers are directly related to the development of TI. Addressing this gap is important because understanding the relationship between student teachers’ interactions with cooperating teachers and their TI can provide practical guidance for training and supporting cooperating teachers. Such knowledge can help optimize practicum supervision, enhance the effectiveness of mentoring in fostering TI, and ultimately support student teachers’ long-term development and commitment to the teaching profession.

### 1.3 Theoretical framework

This study is grounded in Randall Collins’s Interaction Ritual Chain (IRC) theory, a sociological framework widely used to explain how social interactions generate shared meanings, emotions, and identities (Collins, 2014; Hill et al., 2022). This theory conceptualizes the broader system in which interaction rituals take place, specifying their key components, the processes involved in their occurrence, and the outcomes they produce. IRC theory noted that interaction rituals ranging from formal practices such as religious ceremonies to informal exchanges like everyday conversations, constitute the foundation of ongoing social life (Johannessen and Collins, 2024).

According to IRC theory, interaction rituals are defined by several interrelated conditions (Collins, 2014; Duan et al., 2026), including bodily co-presence, which allows participants to perceive and respond to one another directly; boundaries to outsiders, which demarcate participants from non-participants and temporarily establish a distinct interactional group; a mutual focus of attention, whereby participants orient themselves toward a shared activity, object, or event; and a shared mood, understood as a collectively experienced emotional state that develops through interaction. When these conditions converge and are sustained over the course of an encounter, the interaction operates as an effective ritual capable of producing enduring social and emotional outcomes. At the center of interaction ritual chains is the production of identity (Kallio and Törnberg, 2025). Identity formation emerges through participants’ mutual recognition and a high degree of emotional resonance, with the level of emotional energy determining the strength of identity outcomes. Emotional energy is sustained through rhythmically coordinated feedback, which, when effective, contributes to group solidarity by reinforcing participants’ sense of belonging and identification (Zou et al., 2025).

Previous studies on TI development have adopted a variety of theoretical and methodological approaches, including grounded theory (Zhu S., 2025), the cultural-historical activity theory and the dilemmatic space (Zhu G., 2025), the two-component model of attitude (Liu and Keating, 2021) and socio-ecological perspective (Shen et al., 2024). These approaches have provided valuable insights into teachers’ beliefs, attitudes, discursive positioning, and role-related self-understandings. However, they tend to emphasize cognitive processes, individual perceptions, or broader structural influences, while paying comparatively limited attention to the micro-level dynamics of face-to-face interaction and the emotional processes through which identities are enacted and sustained in everyday practice.

In contrast, IRC theory foregrounds interaction itself as the central mechanism of identity construction, explicitly linking emotional energy, mutual recognition, and interactional rhythms to the emergence of identity outcomes. Some studies have also employed the IRC theory to examine identity formation (Cheng et al., 2025; Li and Liang, 2025). This perspective is particularly suited to the practicum context, which is characterized by repeated, face-to-face interactions between PPETs and their cooperating teachers. Applied to this context, IRC highlights that these interactions are both cognitive and emotional in nature (see Table 1). While the exchange of pedagogical knowledge and practical skills is indispensable, the emotional dynamics embedded in these interactions often shape the depth and effectiveness of cognitive engagement (Dreer-Goethe, 2025). Through bodily co-presence, shared focus, and reciprocal recognition, emotional and symbolic resources are exchanged and redefined, generating shared moods and strengthening interactional solidarity over time (Wang et al., 2025). This emergent solidarity provides the social and emotional foundation for the construction of PPETs’ teacher identity.

**Table 1:** Alignment between Collins’ interaction ritual chain and scale domain.

Components of the interactive ritual chain		Dimensions of the interaction
Domains	Bodily co-presence	Duration of interaction
	Barriers to outsiders	Student teachers’ engagement, cooperating teachers’ attitude
	Common action or event/ mutual focus of attention	Cooperating teachers’ attentiveness to student teachers
	transient emotional stimulus/shared mood	Interaction atmosphere
	Feedback intensification through rhythmic entrainment	Frequency of interactions
Ritual outcomes	Group solidarity	Teacher identity

### 1.4 The purpose, questions, and hypothesis of the study

Although the role of cooperating teachers in preservice teacher development has been widely acknowledged, to the best of our knowledge, there was limited research has directly examined how interactions with cooperating teachers relate to the development of TI among PPETs during the teaching practicum (Wei and Chen, 2015). Notably, no empirical studies published since 2022 have explicitly addressed this relationship. To address this gap, the present study poses the following research question: what is the relationship between PPETs’ interactions with their cooperating teachers during the teaching practicum and their TI development? Based on IRC theory, which emphasizes the socially constructed nature of identity through repeated interpersonal interactions, the following hypothesis is proposed: the quality of interactions between PPETs and their cooperating teachers is positively associated with and significantly predicts PPETs’ TI.

## 2 Materials and methods

Although formal Institutional Review Board (IRB) approval is not required in China, this study followed standard IRB principles. No personally identifiable information was collected, and all participants received a cover letter explaining the purpose of the research and highlighting that participation was voluntary and responses would remain confidential.

### 2.1 Participants

Data were collected from December 2024 to February 2025 at 21 PETE programs across China, covering all five regions (i.e., Southern, Northern, Western, Eastern, and Central regions). Participants were recruited through program coordinators who distributed the study information and invitation to all eligible PPETs at the start of their practicum. An online survey link was sent to participants through program coordinators. Before starting the questionnaire, participants were presented with a detailed informed consent form outlining the purpose of the study, potential risks and discomforts, possible benefits, the type of data to be collected, and how their information would be protected. Only those who provided written informed consent were allowed to complete the questionnaire.

Of the 1,250 PPETs initially recruited for the study, 944 were finally retained for analysis after data screening. Among them, 552 (58.5%) were male and 392 (41.5%) were female. Regarding practicum duration, 28 participants (3.0%) reported less than 1 month, 568 (60.2%) reported one to 2 months, 316 (33.4%) reported three to 4 months, and 32 (3.4%) reported more than 5 months. Detailed participant information is presented in Table 2.

**Table 2:** Demographic information of the participants involved in the study.

Demographic variables		*n* (%)
Gender	Female	552 (58.5%)
	Male	392 (41.5%)
Teaching level during practicum	Elementary	345 (36.5%)
	Junior high school	289 (30.6%)
	Senior high school	209 (22.1%)
	University	101 (10.6%)
Practicum duration	Less than 1 month	28 (3%)
	1–2 months	568 (60.2%)
	3–4 months	316 (33.4%)
	More than 5 months	32 (3.4%)
Number of classes taught per week	1–4 class(es)	152 (16.1%)
	5–8 classes	416 (44.1%)
	9–12 classes	284 (30.1%)
	13 or more classes	92 (9.7%)

### 2.2 Instrument

#### 2.2.1 The status of interaction between student teachers and cooperating teachers scale

The original Status of Interaction between Student Teachers and Cooperating Teachers Scale, developed by Wei and Chen (2015) based on IRC Theory, was initially designed for teacher education majors in subjects such as Chinese and English. Subsequently, Xie employed this scale in her master’s thesis to examine interaction patterns between mentors and master’s students in music conservatories (Xie, 2025). Given the specific pedagogical features of physical education, the present study adapted the scale to better reflect the interaction context of student teachers. The revised questionnaire covered demographic information as well as dimensions of interaction with cooperating teachers (e.g., frequency, duration, atmosphere, student teachers’ engagement, cooperating teachers’ attitude, and cooperating teachers’ attentiveness to student teachers). Content validity was assessed by a panel of 12 experts in physical education and teacher education, yielding satisfactory results. Reliability was examined using a two-week test–retest procedure with 50 participants, resulting in a correlation coefficient of *r* = 0.79, indicating acceptable stability of the instrument (McCrae et al., 2011; Hendrik et al., 2025).

#### 2.2.2 Pre-service physical education teachers’ teacher identity scale

The instrument measuring PPETs’ TI was originally developed in Zhang’s (2017) master’s thesis (Zhang, 2017). In the Chinese context, master’s theses are subject to rigorous expert evaluation procedures, often including double-blind review prior to degree conferral, which supports the methodological rigor of the instrument (Ge et al., 2024). Since its development, this instrument has been adopted by several subsequent studies (Chen et al., 2023; Yang et al., 2023; Chen, 2025). Specifically, this instrument includes four domains: value and expectation, perceived confidence of teaching PE, affects and professional growth. The internal consistency coefficient of the full scale was 0.838, and the Cronbach’s alpha value of each dimension is greater than 0.70. It indicated that the scale was acceptable.

### 2.3 Data collection

The questionnaire was administered via the online platform “Wenjuanxing,” a widely used survey tool in China comparable to Amazon Mechanical Turk (Yan et al., 2024). Prior to participation, respondents were informed about the study’s objectives, given clear instructions for completing the questionnaire, and assured of the confidentiality of their responses. Participation was entirely voluntary and anonymous, with no personal identifying information collected to minimize potential response pressure. To enhance data quality, several measures were implemented to reduce response bias, including excluding cases with unusually short completion times, extreme scores, and responses with incorrect codes (Ward and Meade, 2023), and verifying that all valid submissions originated from unique IP addresses to prevent duplicate entries.

### 2.4 Data analysis

All analyses were conducted using IBM SPSS Statistics 26. Descriptive statistics were computed to summarize participants’ demographic characteristics and main study variables. Pearson’s product–moment correlations were then used to assess bivariate associations, with statistical significance determined at the conventional threshold of *p* < 0.05 (Ravid, 2024). Multiple linear regression analyses were performed to test predictive relationships. For each model, unstandardized coefficients (B) with bootstrapped standard errors, standardized coefficients (*β*), *t*-values, and *p*-values were reported (Thomas et al., 2023). Model fit was evaluated using *R*^2^ and adjusted *R*^2^. Statistical assumptions were checked before interpretation. Multicollinearity was assessed via tolerance (> 0.10) and VIF (< 10) (James et al., 2023). Residual independence was tested using the Durbin-Watson statistic, with values between 1.50 and 2.50 indicating no serious autocorrelation (George and Mallery, 2024).

## 3 Results

### 3.1 Correlation analysis of cooperating teacher-student teacher interactions and teacher identity

The Pearson correlation results are presented in Table 3. All six dimensions of cooperating teacher-student teacher interactions were significantly and positively correlated with PPETs’ TI (*p* < 0.01). Among them, interactive atmosphere (*r* = 0.576), cooperating teachers’ attitude (*r* = 0.532), and cooperating teachers’ attentiveness to student teachers (*r* = 0.452) showed stronger associations, suggesting that relational and affective factors of the cooperating teacher played a more critical role than structural aspects of interaction such as frequency (*r* = 0.346) or duration (*r* = 0.321). Notably, student teachers’ engagement (*r* = 0.440) also demonstrated a moderate and significant correlation with TI, underscoring the joint contribution of both parties in shaping TI development.

**Table 3:** Pearson correlation of teacher-student interaction and teacher identity.

Instructor type	Interactive dimensions	Teacher identity	*p*
Cooperating teacher	Frequency of interactions	0.346^**^	0.000
	Duration of interaction	0.321^**^	0.000
	Interactive atmosphere	0.576^**^	0.000
	Degree of student teachers’ engagement	0.440^**^	0.000
	Cooperating teachers’ attitude	0.532^**^	0.000
	Cooperating teachers’ attentiveness to student teachers	0.452^**^	0.000

**p < 0.01, *p < 0.05.

### 3.2 Regression analysis of cooperating teacher-student teacher interactions and teacher identity

Regression results are summarized in Tables 4, 5. The overall model reached statistical significance, with an adjusted *R^2^* of 0.603, indicating that the six interaction dimensions together explained 60.3% of the variance in PPETs’ TI. The Durbin-Watson statistic (1.85) suggested no severe autocorrelation, confirming the appropriateness of the regression model.

**Table 4:** Summary of the model of factors influencing teacher identity.

Model	*R*	*R* ^2^	Adjusted *R*^2^	Errors in standardized estimates	Durbin-Watson
1	0.778^a^	0.606	0.603	8.303	1.85^b^

a Predictor variables: (constant), teacher’s knowledge of students, length of interaction, students’ engagement, number of interactions, teacher’s mentoring attitude, interactive atmosphere.

b Dependent variable: teacher identity.

**Table 5:** Regression model coefficients.

Model		Unstandardized coefficient		Standardized coefficient	*T*	*P*	Statistical analysis of covariance	
		*B*	Boot SE	*β*			Tolerances	*B*
1	(Constant)	37.393	1.661		22.515	0.000		
	Frequency of interactions	3.212	0.482	0.143	6.665	0.000	0.920	1.087
	Duration of interaction	2.756	0.485	0.121	5.682	0.000	0.925	1.082
	Interactive atmosphere	6.547	0.471	0.318	13.901	0.000	0.802	1.246
	Degree of student engagement	4.149	0.415	0.217	10.000	0.000	0.890	1.123
	Cooperating teachers’ attitude	4.525	0.386	0.266	11.717	0.000	0.820	1.220
	Cooperating teachers’ attentiveness to student teachers	3.957	0.405	0.214	9.773	0.000	0.874	1.144

As shown in Table 5, all six predictors exerted significant positive effects. The strongest predictor was interactive atmosphere (*β* = 0.318, *p* < 0.001), followed by cooperating teachers’ attitude (*β* = 0.266, *p* < 0.001). Student teachers’ engagement (*β* = 0.217, *p* < 0.001) and cooperating teachers’ attentiveness (*β* = 0.214, *p* < 0.001) also contributed meaningfully. By contrast, structural dimensions—frequency (*β* = 0.143, *p* < 0.001) and duration of interaction (*β* = 0.121, *p* < 0.001)—were weaker but still statistically significant predictors. These findings indicate that while the intensity of interaction is relevant, the quality and affective tone of the interaction more substantially shape the formation of TI.

## 4 Discussion

### 4.1 Preconditions for interaction: bodily co-presence

A crucial finding relates to the centrality of bodily co-presence between cooperating teachers and PPETs. According to IRC Theory, successful rituals generate solidarity and a sense of belonging through physical proximity, mutual focus of attention, and shared emotional energy (Collins, 2014; Qu et al., 2025). Our findings further demonstrate that bodily co-presence in the practicum functions as a gatekeeping mechanism for professional participation. In school-based practicum settings, particularly within the hierarchical structure of Chinese schools, bodily co-presence becomes pedagogically meaningful only when PPETs are granted opportunities to engage in co-teaching, joint lesson planning, and classroom management (Xu and Lu, 2024). When cooperating teachers actively involve PPETs in instructional practices, co-presence enables the enactment of legitimate teaching roles, generating symbolic inclusion and reinforcing professional belonging (Carey and Simonton, 2025; Dong et al., 2021). Conversely, physical presence without pedagogical engagement results in weakened or incomplete interaction rituals, limiting emotional energy accumulation and constraining identity development. This finding extends IRC theory by highlighting that bodily co-presence must be pedagogically consequential in order to support sustained professional identity formation and underscores the importance of designing practicum programs that ensure sustained bodily co-presence and shared pedagogical action.

### 4.2 Emotional climate and mentoring style as catalysts for identity

Another salient result concerns the emotional climate shaped by cooperating teachers’ mentorship style. A supportive, encouraging, and respectful atmosphere promotes positive emotional energy that bolsters preservice teachers’ self-confidence and commitment to the profession (Valtierra, 2024). Praise, constructive feedback, and empathetic guidance serve as symbolic affirmations that enable PPETs to view themselves as capable and valued future teachers (Rodriguez et al., 2020). In contrast, a critical, dismissive, or authoritarian mentorship style risks creating negative emotional climates that may undermine motivation and professional self-concept (Yuan, 2016; Dong and Aziz, 2024). This duality highlights the performative and affective dimensions of TI formation, reminding us that identity is not simply a cognitive construction but also an emotional and relational process (Wong and Liu, 2024). Importantly, the findings resonate with previous research suggesting that mentorship is most effective when it balances professional guidance with emotional support (Hamman et al., 2006; Davis et al., 2023). From a practical perspective, these findings point to the need for more systematic preparation and support for cooperating teachers. Rather than assuming that effective mentoring emerges naturally from teaching experience, teacher education programs should provide targeted training that helps cooperating teachers develop awareness of the emotional and relational dimensions of mentorship. Such training could focus on constructive feedback strategies, emotionally responsive communication, and reflective mentoring practices that foster positive emotional climates during the practicum.

### 4.3 The importance of student teachers’ active engagement

While cooperating teachers play a central role, the findings also suggest that PPETs’ active participation significantly predicts identity development. Student teachers who immersed themselves in lesson planning, classroom teaching, and reflective dialogue reported higher levels of TI compared with peers who adopted a more passive stance. Preservice teachers’ TI during internships is strongly shaped by their active learning engagement. Although they are formally recognized as members of the teacher community, insufficient engagement may confine them to peripheral participation and impede stable TI formation (Yu et al., 2021; Cai et al., 2022). When PPETs move from peripheral observation to active engagement, they begin to internalize professional norms, develop agency, and negotiate their emerging professional selves (Beijaard et al., 2004). Prior studies in teacher education similarly show that preservice teachers who demonstrate initiative and reflective capacity are better able to integrate practicum experiences into coherent professional identities (Seyri and Nazari, 2023; Le and Bui, 2024). PPETs not as passive recipients of mentoring but as co-producers of interaction rituals. Successful ritual chains depend not only on structural opportunities provided by cooperating teachers but also on preservice teachers’ willingness to invest emotional and cognitive resources. Active engagement thus mediates the relationship between interaction and identity, determining whether practicum experiences contribute to identity consolidation or result in fragmented professional self-concepts. Therefore, physical education teacher education programs should not only facilitate supportive mentorship but also cultivate preservice teachers’ capacity for active involvement and self-directed learning (Mokoena and van Tonder, 2024).

### 4.4 Optimizing the methods and timing of interaction

The study also revealed that the modes and timing of interactions matter considerably. While post-lesson feedback is valuable for identifying strengths and areas for improvement, its retrospective nature limits immediate application (Hyde, 2024). More interactive and participatory forms, such as co-teaching, in-class modeling, and collaborative reflection sessions provide real-time opportunities for preservice teachers to learn through practice and reflection (Kervinen et al., 2022). Such practices enable the integration of theoretical knowledge with practical experience and help preservice teachers internalize professional dispositions. Moreover, diversified interactive strategies reduce the risk of identity foreclosure, where student teachers adopt uncritically the identity of their mentors, as indicated in a master’s thesis by Yang (2021). Instead, varied modes of engagement support critical reflection and the development of personalized professional trajectories. This finding suggests that practicum programs should move beyond unidirectional feedback toward dialogic, experiential, and iterative forms of interaction that sustain long-term identity development. In addition, cooperating teachers should receive targeted training to ensure their guidance is pedagogically aligned and contextually relevant.

### 4.5 Implications

The results of this study have both practical and theoretical implications. These findings suggest that teacher education programs should structure practicum experiences to facilitate frequent, meaningful, and emotionally supportive interactions between cooperating teachers and PPETs. Furthermore, training for cooperating teachers should address not only instructional supervision but also the cultivation of positive relational climates that support the development of TI. Theoretically, the study applies IRC Theory to TI research, demonstrating that ritual elements such as bodily co-presence, mutual focus, and emotional energy can influence the formation of professional identity. Overall, the findings provide empirical support for the relational and affective foundations of TI and integrate sociological and educational perspectives, thereby enhancing understanding of how preservice teachers come to perceive themselves as educators.

### 4.6 Limitations

There are several limitations that should be noted. First, the reliance on self-reported questionnaires may introduce bias, as participants’ perceptions do not necessarily reflect the full complexity of their practicum experiences. In particular, the absence of direct, participatory observation limited our ability to capture the nuanced, situated interactions between student teachers and their cooperating teachers, including the non-verbal, contextual, and affective dimensions that are often overlooked in survey-based research. Second, the cross-sectional design restricts causal inference; longitudinal or mixed-method approaches would provide stronger evidence regarding how TI develops over time. Third, the sample was drawn from a single national context, which may limit the generalizability of findings to other cultural or institutional settings. Future studies could address these limitations by integrating ethnographic observation, adopting longitudinal designs, and including more diverse samples to provide a more comprehensive understanding of TI formation during the practicum. Finally, this study did not collect or analyze information regarding the number of cooperating teachers per school, the degree of interaction between cooperating teachers and pre-service teachers, or the specific roles of cooperating teachers in mentoring or supervising. These factors may influence PPETs’ practical experiences and professional development, and thus represent important directions for future research.

## 5 Conclusion

This study investigated the relationship between cooperating teacher-student teacher interactions and TI development among PPETs during the teaching practicum. The hypothesis proposed that higher-quality interactions with cooperating teachers would be positively associated with, and significantly predict, PPETs’ TI. These results provide clear support for this hypothesis. Both correlation and regression analyses showed that all dimensions of cooperating teacher-student teacher interaction were positively related to PPETs’ TI, with interaction quality explaining a substantial proportion of variance in identity development. Notably, relational and affective dimensions, such as interactive atmosphere, cooperating teachers’ attitudes, and attentiveness were stronger predictors than structural features, including interaction frequency and duration. This pattern suggests that TI formation is shaped primarily by how interactions are experienced, rather than by their mere occurrence. Overall, the findings corroborate the view that TI is constructed through meaningful interpersonal interactions in practicum settings. They highlight the central role of supportive and engaging mentoring practices in fostering PPETs’ TI development.

## Data Availability

The raw data supporting the conclusions of this article will be made available by the authors, without undue reservation.
